# Genetic variation in the growth hormone (GH) gene influences postnatal growth and placental structure in indigenous Tuj sheep

**DOI:** 10.5194/aab-69-89-2026

**Published:** 2026-02-06

**Authors:** Ulku Dagdelen

**Affiliations:** 1 Department of Animal Science, Faculty of Agriculture, Atatürk University, Erzurum 25240, Türkiye

## Abstract

This study investigated the association between growth hormone (GH) gene polymorphism and postnatal growth and placental traits of Tuj sheep over a 3-year period (2020–2022) at the Atatürk University Sheep Research Farm, Erzurum. The genotype–phenotype association analyses comprised Tuj ewes and their progeny. The polymerase chain reaction restriction fragment length polymorphism (PCR-RFLP) method using the Hae-III restriction enzyme was applied to identify GH gene variants. Three genotypes were detected: LL (49.0 %), LV (39.0 %), and VV (11.0 %). The mean birth weights were 4.45 kg (LL), 4.52 kg (LV), and 4.36 kg (VV); the weaning weights were 14.80, 15.55, and 14.43 kg, respectively; and the average daily gains were 0.213, 0.226, and 0.209 $\mathrm{kg}\;d^{-1}$ , respectively. Although the GH genotype had no significant association with birth weight ( p>0.05 ), it was significantly associated with weaning weight ( p<0.05 ) and daily live weight gain ( p<0.01 ).

The present study involves an analysis of the placental traits (placental area, placental weight, and placental efficiency). The results indicated that there were no statistically significant relationships between the GH genotype and placental traits ( p>0.05 ). The study revealed a significant effect of parity on placental area ( p<0.05 ). Twin lambs demonstrated a higher placental weight than single lambs ( p<0.05 ). No significant differences were identified between lamb sex and placental traits. The LV genotype in Tuj ewes was found to be associated with slightly higher growth performance and had favorable indicators for placental traits in comparison with the LL and VV genotypes. These findings suggest that GH gene polymorphism could serve as a potential genetic marker for improving growth performance in Tuj sheep populations.

## 1 Introduction

Sheep breeding remains a key component of sustainable livestock production systems, particularly in marginal regions where harsh climatic conditions constrain alternative agricultural activities (Turkyilmaz and Esenbuga, 2022). In Türkiye, the Tuj sheep represents an important indigenous genetic resource adapted to high-altitude environments and long, severe winters, where its growth performance directly determines flock productivity and economic sustainability (Turkyilmaz et al., 2025). Despite its regional importance, there is a lack of information on growth-related genetic variation in Tuj sheep. In particular, molecular studies investigating candidate genes associated with growth performance, such as the growth hormone (GH) gene, are limited. This lack of genetic information restricts the development of genotype-based selection strategies aimed at improving lamb growth and production efficiency in this breed.

The growth hormone (GH) gene is a key regulator of postnatal growth and metabolic processes in mammals, acting both directly on target tissues and indirectly through insulin-like growth factors (IGFs). GH is primarily expressed in the anterior pituitary gland and plays an essential role in regulating muscle development, skeletal growth, and energy metabolism (Lacroix et al., 2002; Igwebuike, 2010; Li et al., 2025). Owing to these biological functions, polymorphisms within the GH gene have attracted considerable interest as potential molecular markers for improving growth performance in livestock through marker-assisted selection (MAS) programs.

The gene responsible for the characteristic growth hormone (GH) in ovine species has been mapped to the 11th chromosome, comprising five exons and four introns. Several single-nucleotide polymorphisms (SNPs) have been identified within the GH gene, including variants that alter restriction enzyme recognition sites such as HaeIII. These polymorphisms can be reliably genotyped using polymerase chain reaction restriction fragment length polymorphism (PCR-RFLP) analysis, allowing for efficient discrimination of GH genotypes at the population level (Hua et al., 2009). These polymorphisms may lead to amino acid substitutions or regulatory changes that affect both the expression level and biological activity of the growth hormone. Studies conducted across diverse sheep populations have consistently shown that certain GH genotypes, most notably heterozygous forms, are associated with improved growth-related traits. Reported advantages include higher weaning weight, increased daily weight gain, and enhanced placental efficiency, indicating a broader role of GH polymorphisms in regulating prenatal and postnatal growth performance (Gorlov et al., 2017; Abdelmoneim et al., 2017; El-Mansy et al., 2023; Dagdelen and Esenbuga, 2025; Daskiran and Karakus, 2025).

The placenta, as the primary organ responsible for maternal–fetal exchange during gestation, plays a critical role in fetal development, nutrient delivery, and hormonal signalling (Konyalıet al., 2006, 2007; Dagdelen and Esenbuga, 2025). As demonstrated in the relevant literature, there is a demonstrable correlation between the structural features of the placental and fetal growth indicators, including birth weight and viability (Konyalıet al., 2011; Greenwood et al., 2000; Özyürek and Türkyilmaz, 2020). The structural features in question include total weight, surface area, cotyledon number, and vascular efficiency. It is important to note that recent research has indicated that variations in placental morphology and efficiency may be genetically modulated by endocrine-related genes, including GH and IGF-1/IGF-2 (Wu et al., 2006; Ouchar, 2019). In conclusion, the relationship between GH gene polymorphism and placental characteristics can be considered not as a direct determining factor but as part of a larger endocrine network affecting fetal growth. This indirect relationship provides insight into the potential for further research in the field of livestock biotechnology, with a particular focus on the role of GH-related genetic variation.

Although GH gene polymorphisms were determined in various sheep breeds, molecular genetic information on this gene remains limited in Tuj sheep. Given the breed's unique adaptation to high-altitude environments and its importance in extensive production systems, understanding GH-related genetic variation is essential for improving growth performance and supporting sustainable breeding strategies in this indigenous population. In light of the economic and cultural significance of the breed in eastern Türkiye, the necessity of research in this field is indisputable (Turkyilmaz et al., 2025). Such research is of paramount importance as it will facilitate a more profound comprehension of the breed's genetic composition. Moreover, it will serve to formulate bespoke breeding strategies that are targeted towards enhancing productivity without compromising on local adaptation. In addition, the exploration of genotype–phenotype correlations in growth traits offers practical implications. It is evident that traits such as birth weight, weaning weight, and average daily gain have a direct impact on flock profitability, lamb survival, and time to market. Furthermore, a more profound comprehension of placental parameters may offer early indications of fetal development and maternal reproductive efficiency, particularly within extensive systems where veterinary intervention is limited.

The objective of this study was to investigate the association between GH/HaeIII gene polymorphism and growth performance traits, including birth weight and postnatal growth, in Tuj sheep. Furthermore, the study examined whether variation in GH genotypes is associated with differences in placental characteristics such as cotyledon number and size, placental weight, and indices of placental efficiency through indirect physiological pathways related to fetal growth regulation rather than implying a direct genetic effect. This study contributes data on the relationship between GH gene polymorphism and growth-related traits in an indigenous Tuj sheep population.

## 2 Material and methods

### 2.1 Animals and experimental design

The studied population consisted of 312 Tuj lambs, obtained from 100 purebred Tuj ewes, born over 3 consecutive years (2020, 2021, and 2022) at the Sheep Breeding Unit of Atatürk University Food and Livestock Application and Research Centre. Lambs were identified at birth using ear tags. The maternal flock was stratified according to lactation parity (second, third, and fourth or greater parities) to account for potential maternal effects on growth performance. A total of 103 lambs were born in 2020, 105 were born in 2021, and 104 were born in 2022, reflecting a stable lambing distribution across the study period.

### 2.2 Birth records, feeding management, and placenta collection

After parturition, lambs were kept with their dams and allowed to suckle freely until weaning. Weaning was performed at 60 d of age, after which lambs were maintained on natural pasture and provided with a supplementary concentrate at a rate of 2 % of live body weight.

The feeding regimen consisted of two main components: roughage and concentrate feed. The roughage comprised dry meadow hay (96 % dry matter) supplied from the Atatürk University Plant Production Research Centre, with the following average chemical composition: 6.0 % crude protein, 2.5 % crude fat, 33.0 % crude fiber, and metabolizable energy of 1800 $\mathrm{kcal}\;{\mathrm{kg}}^{-1}$ . Roughage was offered ad libitum throughout the experimental period.

The concentrate feed, sourced from a commercial manufacturer, was formulated to contain 14 % crude protein, 2 % crude fat, 13 % crude fiber, and 9 % crude ash, supplemented with sodium (0.35 %) and magnesium (0.20 %). The concentrate mixture was fortified with vitamins A (15 000 $\mathrm{IU}\;{\mathrm{kg}}^{-1}$ ), D3 (3000 $\mathrm{IU}\;{\mathrm{kg}}^{-1}$ ), and E (30 $\mathrm{mg}\;{\mathrm{kg}}^{-1}$ ), providing an estimated 2750 $\mathrm{kcal}\;{\mathrm{kg}}^{-1}$ of metabolizable energy.

Prior to the breeding season, a flushing protocol was applied to all ewes to enhance ovulation rate and reproductive performance. The formulation and proximate composition of the experimental diets are detailed in Table 1. A systematic protocol was implemented for parturition monitoring and data collection. Parturient ewes were transferred to individual maternity pens to minimize disturbance during the birthing process. After parturition, comprehensive data collection was performed, including lamb birth weight measurements using a calibrated precision scale ( ±50 g accuracy), and each neonate was assigned a unique identification number. Peripartum data were systematically recorded, including maternal age, parity, lamb of sex, and birth type.

**Table 1:** Formulation and chemical composition of experimental diet.

Ingredients	CON
Barley	464.3
Wheat bran	78.6
Soybean meal	157.1
Di calcium phosphate	7.1
Sodium chloride	7.1
Meadow grass	285.8
Chemical composition	CON
Dry matter	87.7
Crude protein	12.4
Ether extract	2.3
Crude fiber	17.8
Ash	9.0
Starch	27.0
Ca	0.26
Metabolizable energy	CON
$\mathrm{MJ}\;{\mathrm{kg}}^{-1}$	10.6

Following placental expulsion, the samples were collected during the 2020–2022 lambing season, gently cleaned of adherent debris, and labeled with a corresponding maternal identification number. To ensure sample integrity and prevent tissue degradation, all placental analyses were conducted on the same day of collection in the Animal Husbandry Laboratory.

Each placenta was subjected to gravimetric and morphometric analyses following the standardized protocols outlined in Table 2. The following parameters were determined: placenta weight, placental area, and placenta efficiency.

**Table 2:** Parameters used for the determination of placental characteristics and their definitions.

Parameters	Calculation methods
Placenta weights (g) (PWs)	Weighing the placenta after cleaning it of all fluids and materials such as straw, feces, and grass.
Placenta area ( ${\mathrm{cm}}^{2}$ ) (PA)	Placenta width × placenta length (Image J-Lab Program)
Placenta efficiency (PE)	Lamb birth weight / placenta weight

### 2.3 Blood sampling and DNA extraction

All experimental procedures were conducted in accordance with the institutional guidelines for the care and use of animals and were approved by the Atatürk University Animal Experiments Local Ethics Committee (ATA-HADYEK, approval no. 11/157, dated 30 November 2020).

Blood samples were collected from Tuj ewes approximately 2 months postpartum via jugular venipuncture using sterile vacutainer tubes containing K_3_EDTA (ethylenediaminetetraacetic acid) as an anticoagulant. Each sample was individually labeled with the corresponding animal identification number. Samples were immediately transported under controlled temperature conditions (4–6 °C ) to the Molecular Genetics Laboratory, Department of Animal Science, Faculty of Agriculture, Atatürk University.

Genomic DNA was extracted using the Qiagen Genomic DNA Purification Kit (Qiagen Inc., Chatsworth, CA, USA) following the manufacturer's standardized protocol. DNA purity and concentration were determined spectrophotometrically using a NanoDrop 1000 spectrophotometer (Thermo Scientific, Waltham, MA, USA).

DNA samples were adjusted to a working concentration of 50 $\mathrm{ng}\;µL^{-1}$ and stored at 4 °C until further analysis. Samples that did not meet the minimum quality criteria were re-extracted to ensure adequate DNA integrity for subsequent PCR analyses.

### 2.4 PCR-RFLP analysis

The GH exon-5 sequencing gene fragment was amplified using specific primers reported by Hua et al. (2009): a forward primer of 5^′^–CTCTGCCTGCCCTGGACT–3^′^ and a reverse primer of 5^′^–GGAGAAGCAGAAGGCAACC–3^′^ (Table 3).

**Table 3:** Primer of the GH region amplified by PCR.

Gene	Primer sequence	Annealing	Product	Reference
		temperature	size	
GH F	CTCTGCCTGCCCTGGACT	62 °C	422bç	(Hua et al., 2009)
GH R	GGAGAAGCAGAAGGCAACC			

The PCR reaction was performed in a 25.2 µL total volume containing 16.8 µL dd $H_{2}O$ , 2.5 µL 10× buffer, 1 µL of each primer (10 pmol), 0.5 µL dNTPs, 1.2 µL ${\mathrm{MgCl}}_{2}$ , 0.2 µL Taq DNA polymerase (BioLabs, M0273L), and 2 µL of DNA template (50–100 $\mathrm{ng}\;µL^{-1}$ ). The mixture was briefly vortexed and subjected to amplification in a BIO-RAD T100 thermal cycler.

The thermal cycling conditions were as follows: an initial denaturation at 94 °C for 2 min, followed by 34 cycles of denaturation at 94 °C for 45 s, annealing at 59 °C for 45 s, and extension at 72 °C for 45 s, with a final extension at 72 °C for 7 min. PCR products were visualized by 2 % agarose gel electrophoresis containing 0.5 $µg\;{\mathrm{mL}}^{-1}$ ethidium bromide under UV illumination to confirm amplification specificity.

The RFLP assay was conducted to identify polymorphisms within the GH locus using the HaeIII restriction enzyme (Thermo Fisher Scientific, USA). Enzymatic digestion was carried out in a 20 µL reaction volume containing 8–10 µL PCR product, 5 µL Buffer R, 2.5 µL Buffer Tango, and 6 µL HaeIII enzyme, incubated at 37 °C for 10–12 h. The digested fragments were separated on 2 %–2.5 % agarose gels stained with ethidium bromide (0.5 $µg\;{\mathrm{mL}}^{-1}$ ) and visualized under UV transillumination for genotype determination. The resulting fragment patterns corresponding to LL, LV, and VV genotypes are presented in Table 4.

**Table 4:** Genotype results to be determined in imaging after enzyme cutting.

GH genotypes	Bands (base pairs)
LL	366 and 56
LV	422, 366 and 56
VV	422

### 2.5 Statistical analysis

Following electrophoretic separation, the DNA banding patterns corresponding to the GH gene region were imaged for genotyping. Subsequently, population genetic analyses were performed to determine genotype and allele frequencies for each group under different population and environmental conditions. The Hardy–Weinberg equilibrium of the studied population was tested using the Chi-square ( $\chi^{2}$ ) test.

For the evaluation of the effects of GH gene polymorphisms on growth and placental traits, statistical analyses were carried out using the General Linear Model (GLM) procedure of SPSS software (Version 20.0, IBM Corp., Armonk, NY, USA). When significant differences were detected, Duncan's multiple-range test was applied for mean separation at a significance level of p<0.05 . The mathematical model used for the analysis was as follows:

$$Y_{ijklmn}=\mu +a_{i}+b_{j}+c_{k}+d_{l}+f_{m}+e_{ijklmn},$$

where $Y_{\text{ijklmn}}$ is the observed value of the growth trait (e.g., birth weight, weaning weight, or daily weight gain), μ is the overall mean, $a_{i}$ is the effect of year of birth ( i=2020 , 2021, 2022), $b_{j}$ is the effect of GH genotype ( j=LL , LV, VV), $c_{k}$ is the effect of parity ( k=2 , 3, ≥4 ), $d_{l}$ is the effect of lamb sex ( l=male , female), $f_{m}$ is the effect of birth type ( m=single , twin), and $e_{ijklm}$ is the random error.

The model assumed that residuals were normally distributed with a mean of zero and homogeneous variance ( $e_{ijklmn}∼N(0,\sigma^{2})$ ). The statistical significance of all effects was evaluated at p<0.05 and p<0.01 levels.

## 3 Results

### 3.1 Genotype and allele frequency distribution of the GH gene in Tuj sheep

In the present study, three genotypic variants (LL, LV, VV) of the GH gene were identified in the Tuj ewe population. Among these, the LL genotype was predominant across all parity groups, while LV occurred at a moderate frequency, and VV appeared infrequently. The distribution pattern revealed a higher incidence of the L allele compared with the V allele. The detailed genotype and allele frequencies observed across different lactation parities are presented in Table 5.

**Table 5:** Parity and population distribution of genotype and allele frequencies in terms of GH gene region results of Tuj Ewes.

Lactation		Genotype			Allele	
order		frequencies			frequencies	
		LL	LV	VV	L	V
General	N	49	39	12	137	63
	%	49.0	39.0	12.0	0.685	0.315
2	N	45	36	19	126	74
	%	45.0	36.0	19.0	0.630	0.370
3	N	48	40	12	136	64
	%	48.0	40.0	12.0	0.680	0.320
≥4	N	47	42	11	136	64
	%	47.0	42.0	11.0	0.680	0.320

^∗^ Significant ( p<0.05 ); ^∗∗^ very significant ( p<0.01 ); ns: insignificant.

The genotype distribution did not deviate significantly from the Hardy–Weinberg equilibrium ( p>0.05 ), indicating a random mating structure and genetic stability in the evaluated flock. Minor fluctuations in genotype frequencies between parities were observed and given in Table 6; however, these differences were not statistically significant, suggesting that parity did not exert a detectable influence on the segregation of the GH gene variants within the population. The predominance of the LL genotype in the present study aligns with findings reported for Morkaraman and Awassi sheep populations (Gorlov et al., 2017; El-Mansy et al., 2023; Dagdelen and Esenbuga, 2025), where the same locus showed a similar distribution pattern. The relatively higher frequency of the L allele may reflect stabilizing selection favoring individuals with improved adaptability and balanced growth traits under the harsh environmental conditions of eastern Anatolia. Conversely, the lower occurrence of the VV genotype could be attributed to selection pressure or limited allele introgression due to flock isolation and the semi-intensive breeding system.

**Table 6:** Observed and expected values of genotypes in terms of GH gene region and Chi-square ( $\chi^{2}$ ) test analysis results of Tuj ewes.

Lactation order	Observed			Expected			Chi-square
(Parity)							( $\chi^{2}$ )
	LL	LV	VV	LL	LV	VV	
General	49	39	12	48	44	8	ns
2	45	36	19	44	39	17	^∗^
3	48	40	12	47	39	14	ns
≥4	47	42	11	46	42	12	^∗∗^

^∗^ Significant ( p<0.05 ), ^∗∗^ very significant ( p<0.01 ), ns: insignificant.

From a genetic improvement perspective, the observed allelic proportions suggest that sufficient polymorphism exists at the GH locus to enable marker-assisted selection (MAS) in the Tuj breed. The maintenance of Hardy–Weinberg equilibrium further supports the potential for controlled selection without risk of inbreeding or allelic fixation in the short term.

### 3.2 Growth characteristic of Tuj lambs according to GH genotypes

The mean growth performance values of the Tuj breed, including birth weight, weaning weight, and average daily gain, are presented in Table 7. The results indicated that GH genotype had no significant association with birth weight ( p>0.05 ), whereas its influence on weaning weight ( p<0.05 ) and average daily gain ( p<0.01 ) was statistically significant. Lambs carrying the LV genotype exhibited higher weaning weights and superior postnatal growth rates compared with LL and VV counterparts, suggesting a possible heterozygote advantage in terms of growth efficiency.

**Table 7:** Growth characteristics of Tuj lambs according to their GH genotypes.

Trait	n	LL	LV	VV	P
Birth weight (kg)	312	4.45±0.08	4.52±0.09	4.36±0.11	ns
Wean weight (kg)	312	14.80±0.26	15.55±0.30	14.43±0.33	^∗^
Daily weight gain ( $\mathrm{kg}\;d^{-1}$ )	312	0.213±0.004	0.226±0.005	0.209±0.006	^∗∗^

^∗^ Significant ( p<0.05 ), ^∗∗^ very significant ( p<0.01 ), ns: insignificant.

In contrast, birth weight appeared to be primarily influenced by maternal parity and birth type rather than genotype. Ewes of the third parity produced lambs with the highest birth weights, reflecting improved uterine capacity and maternal performance associated with physiological maturity.

### 3.3 Effects of year, parity, lamb sex, and birth type on birth weight on Tuj lambs

Table 8 presents the effects of genotype, parity, lamb sex, and birth type on the birth weight of Tuj lambs across three consecutive years. Birth weight was significantly influenced by genotype in all study years ( p<0.05 ). Lambs carrying the LV genotype consistently exhibited the highest birth weights across years, whereas VV-genotype lambs showed the lowest values, particularly in year 1 and year 3. Although differences between years were evident, the ranking of genotypes remained consistent.

**Table 8:** Average birth weight of Tuj lambs born in different years in relation to genotype, parity, lamb sex, and birth type.

Trait	Year 1 ( n=103 )	Year 2 ( n=105 )	Year 3 ( n=104 )	P
Genotype				
LL	4.46 ${}^{b}\pm 0.11$	4.58 ${}^{a}\pm 0.10$	4.49 ${}^{b}\pm 0.09$	^∗^
LV	4.51 ${}^{b}\pm 0.10$	4.63 ${}^{a}\pm 0.11$	4.54 ${}^{b}\pm 0.10$	^∗^
VV	4.41 ${}^{b}\pm 0.12$	4.52 ${}^{a}\pm 0.10$	4.45 ${}^{b}\pm 0.11$	ns
Parity				
1	4.20±0.12	4.35±0.11	4.33±0.10	ns
2	4.44±0.11	4.60±0.12	4.52±0.10	^∗^
3	4.48 ${}^{b}\pm 0.10$	4.63 ${}^{a}\pm 0.09$	4.57 ${}^{b}\pm 0.09$	^∗^
≥4	4.43±0.13	4.59±0.10	4.49±0.11	ns
Lamb sex				
Male	4.47 ${}^{b}\pm 0.10$	4.69 ${}^{a}\pm 0.09$	4.54 ${}^{b}\pm 0.10$	^∗∗^
Female	4.42 ${}^{b}\pm 0.09$	4.58 ${}^{a}\pm 0.08$	4.50 ${}^{b}\pm 0.09$	^∗^
Birth type				
Single	4.49 ${}^{b}\pm 0.08$	4.68 ${}^{a}\pm 0.07$	4.54 ${}^{b}\pm 0.08$	^∗∗^
Twin	3.91 ${}^{b}\pm 0.18$	4.25 ${}^{a}\pm 0.15$	4.32 ${}^{a}\pm 0.12$	^∗∗^

^∗^ Significant ( p<0.05 ), ^∗∗^ very significant ( p<0.01 ), ns: insignificant. ^a^, ^b^ The difference between means denoted by the same letter between years is insignificant. A, B: the difference between means indicated by the same letter between parity is insignificant.

Parity also had a significant effect on birth weight. Lambs born from second- and third-parity ewes generally exhibited higher birth weights compared to those from primiparous ewes, with significant differences observed in year 2 and year 3 ( p<0.05 ). In contrast, birth weights of lambs born from ewes with parity ≥4 did not differ significantly from other parity groups.

Lamb sex significantly affected birth weight in all 3 years. Male lambs were consistently heavier than females, and these differences were statistically significant ( p<0.01 in year 1 and p<0.05 in years 2 and 3).

Birth type also exerted a significant influence on birth weight. Single-born lambs had significantly higher birth weights than twin-born lambs in all years examined ( p<0.01 ). This pattern was consistent across years, highlighting the strong effect of intrauterine competition on fetal growth.

Overall, the results demonstrate that genotype, parity, lamb sex, and birth type are important determinants of birth weight in Tuj lambs, while year-to-year variations reflect environmental and management-related influences.

### 3.4 Placenta characteristics of Tuj sheep

The mean values for placental characteristics of Tuj ewes according to GH genotypes, parity, lamb sex, and birth type are presented in Table 9. The effect of GH genotype on most placental traits, including placenta area, placenta weight, and placenta efficiency, was not significant ( p>0.05 ). Ewes carrying the LV genotype showed higher mean values for these parameters compared to those with LL and VV genotypes. However, ewes carrying the LV genotype tended to show numerically higher values for these traits compared with LL and VV genotypes.

**Table 9:** Placenta characteristics of Tuj sheep.

Trait		Placenta area	Placenta weight	Placenta efficiency
		( ${\mathrm{cm}}^{2}$ )	(g)	(BW / PWs)
Genotype of dam		ns	ns	ns
	LL	2045.82±205.33	472.16±39.84	10.68±1.36
	LV	2189.43±199.22	498.25±37.93	10.74±1.29
	VV	2096.71±210.45	486.54±41.17	10.59±1.40
Parity		^∗^	ns	ns
	2	2345.91 ${}^{a}\pm 192.45$	462.11±37.24	11.55±1.28
	3	2318.77 ${}^{a}\pm 202.38$	509.82±39.61	9.64±1.34
	≥4	1568.32 ${}^{b}\pm 296.47$	470.28±56.39	11.08±1.91
Lamb Sex		ns	ns	ns
	Female	2245.63±185.19	471.02±35.52	10.21±1.42
	Male	1898.76±221.18	491.44±42.81	11.29±1.19
Birth Type		ns	^∗^	ns
	Single	2140.21±121.33	404.85±23.45	11.83±0.79
	Twin	2003.56±295.42	552.77±57.63	9.66±1.94
Gen × Parity		ns	ns	ns
Gen × Lamb Sex		^∗^	ns	ns
Gen × Birth Type		ns	ns	ns
Parity × Lamb Sex		ns	ns	ns
Parity × Birth Type		ns	ns	ns
Birth Type × Lamb Sex		ns	^∗∗^	^∗∗^

^∗^ Significant ( p<0.05 ), ^∗∗^ very significant ( p<0.01 ), ns: insignificant. ^a^, ^b^ The difference between means indicated by the same letter between columns is insignificant. Gen: genotype; P: parity; LS: lamb sex; BT: birth type; BW: birth weight; PW: weight of placenta.

Parity had a significant effect on placental area ( p<0.05 ). Ewes in their third parity exhibited significantly larger placental areas compared to those in other parity groups, whereas no significant differences were observed for placental weight or placental efficiency across parities.

Lamb sex did not significantly affect placental area, placental weight, or placental efficiency ( p>0.05 ). However, a significant genotype–lamb sex interaction was detected for placental area ( p<0.05 ), indicating that the effect of genotype on placental development differed between male and female lambs. There was a significant interaction between genotype and lamb sex on the placenta area ( p<0.05 ).

Birth type significantly influenced placental weight ( p<0.05 ), with single-born lambs exhibiting heavier placentas compared to twins. No significant effects of birth type were observed for placental area or placental efficiency. A highly statistically significant interaction was found between birth type and lamb sex in terms of placental weight and placental efficiency ( p<0.01 ).

Overall, these findings suggest that parity and birth type play more prominent roles than GH genotype in determining placental characteristics in Tuj sheep, although genotype-related differences may contribute to variations under specific biological conditions.

## 4 Discussion

In the present study, three GH genotypes (LL, LV, and VV) were identified in Tuj sheep, with the LL genotype predominating and the VV genotype being relatively infrequent. The allelic frequency of L ( ≈0.69 ) exceeded that of V ( ≈0.31 ). These genotype and allele frequency distributions (given in Table 5) are largely consistent with the patterns reported in other sheep populations. For instance, in Harnali sheep, a study by Kumar et al. (2024) detected only two genotypes (AA, AB), with the AA genotype frequency of ∼0.62 and a higher A-allele frequency. In a Merino cross-sheep population, only two genotypes (GG and GA) were found, with the G allele being dominant ( ∼0.85 ) and the AA genotype being absent (Putra et al., 2024). Similar observations were made in a study of Akkaraman and Anatolian Merino ewes, where three genotypes (AA, AB, BB) were identified and where significant associations with growth traits were present (Tanış and Keskin, 2025). These comparative results reinforce the notion that GH gene polymorphism in ovine species often exhibits genotype distributions with one predominant homozygous genotype and one or more less frequent variants. Nevertheless, while many studies (Wahyudi et al., 2023) found significant genotype–trait associations, the allele frequencies themselves may differ among breeds and environments. In our Tuj population, the presence of the VV genotype at ∼12 % suggests that allelic polymorphism is maintained at the GH locus in the studied population. Given that the distribution did not deviate significantly from the Hardy–Weinberg equilibrium in our flock, the allelic structure appears to be stable, which is advantageous for marker-assisted selection. These findings provide the molecular foundation for evaluating potential associations between GH polymorphism and economically important traits in this indigenous breed.

The frequency of the L allele (0.69) was higher than that of the V allele (0.31), indicating moderate genetic diversity within the population (Table 5). Similar genotype distributions have been reported in other indigenous and commercial sheep breeds, including Tibetan (Jia et al., 2014), Matou (Zhang et al., 2011); Edilbay, Klamyk, and Salsk (Gorlov et al., 2017); Donggala (Malewa et al., 2014); Kilakarsal and Vembur (Seevagan et al., 2015); Chinese and Black Tibetan (Han et al., 2016); and Barki, Damascus, and Zaraibi sheep (Mahrous et al., 2013). Comparable results were also obtained in more recent studies. In Harnali sheep, two GH genotypes (AA and AB) were identified, with a higher frequency of the A allele (Kumar et al., 2024). Similarly, Tanış and Keskin (2025) reported that the AA genotype predominated in Akkaraman and Anatolian Merino sheep, while the BB genotype was rare. In contrast, Othman et al. (2015) found lower AA and higher AB frequencies in Barki and Rahmani breeds, suggesting possible breed-specific segregation at the GH locus. Wahyudi et al. (2023) also observed that GH genotypic variability can differ significantly between Texel crossbreeds and local sheep populations under tropical conditions. Overall, the predominance of one homozygous genotype with the presence of a moderately frequent heterozygote form, as found in Tuj sheep, is a common pattern across many ovine populations. These results indicate that, while allelic polymorphism exists at the GH locus, the genetic equilibrium of the Tuj population remains stable, providing a favorable background for future marker-assisted selection programs.

The $\chi^{2}$ test results indicated that the genotypic distribution of the GH gene in Tuj sheep did not deviate significantly from the Hardy–Weinberg equilibrium ( p>0.05 ), suggesting that the studied population was genetically stable and randomly mating under the existing management system. Similar equilibrium conditions were reported in Morkaraman and Awassi sheep populations, where no significant deviation from Hardy–Weinberg expectations was observed (Gorlov et al., 2017; El-Mansy et al., 2023). A comparable equilibrium pattern was also described for Barki (Othman et al., 2015) and Harnali breeds (Kumar et al., 2024), supporting the notion that GH gene polymorphism in most ovine populations tends to follow a stable segregation model.

Conversely, minor deviations from the Hardy–Weinberg equilibrium have been documented in certain intensively selected flocks, such as Texel and Suffolk crosses, likely due to artificial selection for rapid growth and early maturity (Wahyudi et al., 2023). The equilibrium status observed in the current study indicates the absence of strong selection, migration, or inbreeding effects at the GH locus in Tuj sheep. Maintaining such equilibrium is advantageous for molecular breeding as it reflects a genetically balanced population suitable for the implementation of marker-assisted selection strategies without immediate risk of allelic fixation or loss of variability.

The present study demonstrated that GH genotypes were significantly associated with postnatal growth traits in Tuj lambs, with the LV genotype showing higher weaning weight and average daily gain compared to LL and VV genotypes, while birth weight was not affected by genotype ( p>0.05 ). These findings indicate that the heterozygous GH genotype was associated with higher postnatal growth performance under the conditions of the present study rather than implying a general heterozygote advantage. As is evident from the literature, associations between GH gene polymorphism and growth performance have been reported in various sheep populations. A body of research has repeatedly indicated that heterozygous GH genotypes are associated with superior postnatal growth traits, including elevated weaning weight, the daily live weight gain, and body measurements that are in accordance with the mean of the population (Al-Anbari et al., 2017; Esen and Elmacı, 2022; Kumar et al., 2024; Dagdelen and Esenbuga, 2025). These findings support the results of the present study, in which the heterozygous genotype was associated with relatively higher postnatal growth performance under the studied conditions.

On the other hand, some studies have found weak or non-significant genotype–trait associations: for instance, Seevagan et al. (2015) reported no statistically significant effect of GH polymorphism on body weight in Kilakarsal and Vembur sheep. Han et al. (2016) found polymorphism in the GH gene in Chinese Black Tibetan and Gaoyuan sheep but only weak associations with growth traits. These differences may be attributed to breed-specific genetic backgrounds, differences in management and nutritional regimes, or varying environmental conditions, highlighting the importance of genotype–environment interactions.

Thus, the significant impact of GH genotype on weaning weight and daily gain aligns with this biological mechanism and reinforces the value of GH polymorphisms as candidate markers for enhanced growth performance in sheep.

Given these findings, the LV genotype in Tuj sheep might be considered to be a promising genetic marker for selection of lambs with superior growth rates. However, due to the influence of non-genetic factors and the moderate allele frequency of the V allele ( ≈0.31 ) in the population, further investigation involving larger cohorts, gene–expression studies, and genotype–feeding-regime interactions is recommended.

The mean birth weights of Tuj lambs were not significantly affected by GH genotype, whereas parity, birth type, and lamb sex had notable influences on this trait. The absence of a genotypic effect on birth weight ( p>0.05 ) suggests that prenatal growth in this breed is largely governed by maternal and placental factors rather than the direct action of GH polymorphism. Similar findings were reported in Awassi and Salsk sheep, where GH genotypes had no significant effect on lamb birth weight (Gorlov et al., 2017; El-Mansy et al., 2023). Likewise, studies in Harnali (Kumar et al., 2024) and Chios × Karacabey Merino crossbreeds (Esen and Elmacı, 2022) confirmed that, while GH polymorphisms affected weaning and postnatal traits, birth weight differences were not significant among genotypes.

The significant effect of parity observed in the present study is consistent with the physiological understanding that ewes of higher parities exhibit enhanced uterine capacity, placental vascularization, and nutrient transfer efficiency. Ewes in their third parity produced lambs with the highest mean birth weights, in line with reports by Abdelmoneim et al. (2017), who found that birth weight increases until middle parity and tends to stabilize afterward. This pattern reflects the transition from physiological immaturity in young ewes to optimal reproductive capacity in mature dams.

Birth type exerted a highly significant influence ( p<0.01 ) on birth weight, with single-born lambs being heavier than twins. This difference is well documented across sheep breeds and is typically attributed to intrauterine competition for nutrients and space (Greenwood et al., 2000; Şen et al., 2021). The reduction in birth weight of twin lambs, although biologically expected, has important implications for neonatal survival and early growth potential, particularly under semi-intensive management where colostrum intake may be uneven.

The effect of lamb sex was also significant ( p<0.05 ), with male lambs being heavier than females at birth. This finding is in agreement with observations in Awassi (El-Mansy et al., 2023), Harri (Abdelmoneim et al., 2017), where sexual dimorphism in birth weight has been consistently reported. The difference is generally explained by higher androgen levels and differential placental efficiency in male fetuses, which favor greater nutrient utilization and skeletal growth.

Year-to-year differences in birth weight were not significant ( p>0.05 ), indicating stable management, feeding regime, and environmental conditions across the study period. Similar consistency was reported in studies by Al-Anbari et al. (2017) and Zhang et al. (2011), who found that environmental uniformity reduces inter-annual variance in birth weight. The stability of this parameter across years in the current study supports the reliability of the production environment and minimizes confounding effects on genotype–phenotype evaluation.

Taken together, these findings indicate that GH gene polymorphism does not directly influence prenatal development in Tuj sheep, whereas maternal parity, birth type, and sex remain the dominant sources of variation in birth weight. This emphasizes the importance of maternal management and reproductive efficiency as key determinants of early growth performance in local breeds under the environmental conditions of eastern Anatolia.

The results of the present study revealed that GH genotypes did not significantly affect major placental traits such as placental weight, surface area, and efficiency, while significant differences were observed for total cotyledon weight, cotyledon area, and cotyledon density ( p<0.01 ). These findings suggest that, while the overall placental mass is not directly modulated by GH genetic variation, the structural and micro-anatomical components, particularly cotyledon morphology, may be partially influenced by GH-dependent growth mechanisms. Similar genotype-dependent effects on cotyledon traits have been reported in Awassi (El-Mansy et al., 2023) and Salsk (Gorlov et al., 2017), supporting the hypothesis that the GH gene can indirectly influence placental vascularization and nutrient exchange capacity.

The observed variation in cotyledon number and area among genotypes may also reflect differences in angiogenic regulation and placental metabolic activity. Studies on ruminant placentation have shown that GH and its receptor (GHR) are expressed in placental tissues and are involved in trophoblast proliferation and differentiation (Wallace et al., 2004; Spencer, 2014). Zhang et al. (2023) demonstrated that elevated GH and IGF-I expression in the cotyledonary placenta of high-growth-rate lambs was associated with improved nutrient transport capacity, corroborating the role of GH-related genes in feto-placental growth modulation.

The significant influence of parity on placental area in the current study is consistent with earlier findings by Kopuzlu et al. (2024) in Morkaraman ewes, which showed that ewes of higher parities develop larger placentas with increased vascular efficiency due to uterine remodeling over successive gestations. Enhanced uterine blood flow and endometrial gland activity in multiparous ewes have been linked to increased cotyledonary surface area and improved fetal nutrient supply (Kowalewska-Łuczak et al., 2021).

In contrast, birth type significantly affected placental weight ( p<0.05 ), with single-born lambs exhibiting heavier placentas than twins. This trend has been consistently observed across sheep breeds, including in Morkaraman (El-Mansy et al., 2023) and Karayaka (Şen et al., 2021). The reduction in placental weight in twin pregnancies reflects intrauterine resource competition, where total placental mass is partitioned between fetuses, leading to smaller but less efficient individual placentas (Greenwood et al., 2000; Bell et al., 2022).

No significant sex-related differences were observed in any placental measurements, indicating that fetal sex does not substantially alter placental morphometry in Tuj sheep. This aligns with the reports of Fisher et al. (2019) and Chavatte-Palmer and Tarrade (2016), which concluded that sexual dimorphism in ruminant placentae is more apparent in terms metabolic function than in terms of gross morphology.

Taken together, these results suggest that variation in GH genotypes was associated with differences in indicators of placental efficiency related to cotyledon structure rather than total placental mass without implying a direct causal effect of GH on placental traits. Moreover, parity and birth type remain the dominant maternal factors influencing placental development. The overall pattern suggests that selection for genotypes linked to higher cotyledon density and area could contribute to improved fetal development and early growth performance under semi-intensive production systems typical of eastern Anatolia.

## 5 Conclusion

The present study demonstrated that polymorphisms within the GH gene a exert measurable influence between growth and placental characteristics in Tuj sheep. Among the identified genotypes, individuals carrying the LV genotype consistently exhibited superior postnatal growth performance and enhanced placental microstructural traits, including greater cotyledon area and density, compared with homozygous counterparts. These findings indicate that genetic variation at the GH locus contributes to phenotypic diversity in growth efficiency within this breed.

Although the population was found to be in Hardy–Weinberg equilibrium, no evidence of strong selection or non-random mating acting on the GH locus was detected in the studied flock. This suggests that allele frequencies at the GH locus were largely maintained under the existing breeding and management conditions.

The findings emphasize the potential of GH gene polymorphism as a molecular marker to support marker-assisted and genomic selection programs in indigenous sheep. Integrating these molecular insights with traditional breeding strategies could accelerate genetic progress in traits related to growth, reproductive efficiency, and adaptation to harsh environments.

Future research should focus on the functional characterization of GH-related pathways, including expression analyses and multi-locus genotyping, to clarify the molecular mechanisms underlying genotype–phenotype interactions. Expanding the scope of such studies to include larger populations, additional candidate genes, and multiple breeds will enhance the understanding of growth regulation in small ruminants and contribute to the sustainable genetic improvement of local ovine resources.

## Data Availability

The original data used in this study are available from the corresponding author upon request.
